# Selected Aspects of Navigation System Synthesis for Increased Flight Safety, Protection of Human Lives, and Health

**DOI:** 10.3390/ijerph17051550

**Published:** 2020-02-28

**Authors:** Milan Džunda, Peter Dzurovčin, Ivan Koblen, Stanislav Szabo, Edina Jenčová, Peter Čekan, Peter Korba, Ladislav Főző, Lucia Melníková, Alica Tobisová, Daniel Blaško, Jozef Galanda

**Affiliations:** 1 Department of Air Transport Management, Faculty of Aeronautics, Technical University of Kosice, 041 21 Kosice, Slovakia; milan.dzunda@tuke.sk (M.D.); peter.dzurovcin@tuke.sk (P.D.); stanislav.szabo@tuke.sk (S.S.J.); peter.cekan@tuke.sk (P.Č.); peter.korba@tuke.sk (P.K.); ladislav.fozo@tuke.sk (L.F.); lucia.melnikova@tuke.sk (L.M.); alica.tobisova@tuke.sk (A.T.); daniel.blasko@tuke.sk (D.B.); Jozef.Galanda@tuke.sk (J.G.); 2 Airworthiness Department, Civil Aviation Division, Transport Authority, M.R.Štefánik Airport (BTS), 823 05 Bratislava, Slovakia; ivan.koblen@nsat.sk

**Keywords:** flight safety, flying objects, accuracy and resistance to interference

## Abstract

Accurate navigation systems allow us to optimize the trajectory of flying objects and thus solve environmental problems in aviation and their impact on public health. In this paper, we present one of the methods of assessment of accuracy and resistance to interference of distance-measuring equipment (DME). By using computer technology, the method enables us to determine the potential but also the real error measuring the distance of the flying object from DME. The credibility of the respective results of the solution on the task of DME optimal rangefinder synthesis depends on the accuracy of the previous data used, i.e., mathematical models of the respective flying objects flight dynamics, useful signals, and their parameters and interference. DME systems have an impact on air transport safety, and therefore the impact of interference on their operation must be investigated.

## 1. Introduction

A solution to the problems of reducing harmful greenhouse gas emissions from aviation is currently very actual. In June 2019, the President of the European Council Donald Tusk and the President of the European Commission Jean-Claude Juncker confirmed, in the context of the United Nations Climate Summit and the United Nations Climate Conference (COP25), that the EU will meet the Paris climate change objectives. In the document: “Joint letter of Presidents Donald Tusk and Jean-Claude Juncker on the upcoming G20 summit” states: “We need to leave a healthier planet behind for those that follow. At home, the EU is proposing ambitious targets for reducing CO_2_ emissions by 2030 that are both scientifically accurate and politically indispensable.”

We assume that one of the ways to reduce CO_2_ emissions in aviation is to use new technologies to optimize the flight trajectories of flying objects, thereby reducing harmful gas emissions. Therefore, we have published some of the results of our research in this article. Mark Hertsgaard also talks about the relationship between man and his view of the importance of new technologies for solving the problems of climate sustainability.

We were inspired to write this paper by Mark Hertsgaard, who said: “Technology, of course, lies at the heart of man’s relationship with the environment.” It turns out that without new technologies, environmental problems in air transport are unsolvable. 

In [1], the authors report that carbon dioxide emissions in the atmosphere have negative effects on humanity. Based on this, they propose new approaches to the evaluation of anthropogenic processes. In [2], it is stated that problems related to the greenhouse effect are solved in many scientific works. Their contribution presents one of the possible approaches to solving this problem, which is based on the creation of a new model of combined biogeochemical cycles of carbon and methane. In [3], the authors report that climate change is caused by increased greenhouse gases concentrations. They recommend, inter alia, that CO_2_ emissions should be reduced.

The European Union seeks to maintain Europe’s leading position in international aviation and to increase the efficiency of airspace use. Quality air transport affects the EU economy and the achievement of its climate objectives. DME-type navigation systems allow us to optimize the flight trajectory of flying objects, thereby addressing environmental issues in aviation and their impact on public health. Aviation greenhouse gas emissions have more than doubled over the past two decades. Emissions from international aviation and shipping represent less than 3.5% of total greenhouse gas emissions in the EU, which contribute to climate change, however their emissions have grown in recent years at the fastest pace. This is mainly due to a record increase in the air traffic. Today many more people fly than they used to before, and many more goods are transported.

Before the COP25 summit in Madrid, the European Parliament adopted a resolution calling on the EU to be more ambitious in reducing emissions from aviation and shipping. According to Transport and Environment, Ryanair is a major carbon dioxide producer in Europe. The company’s CO_2_ emissions increased by 6.9 percent in one year. Efforts to reduce carbon dioxide emissions in aviation require airlines to monitor and report CO_2_ emissions. This obligation should apply from 2021. It is assumed that without effective measures, CO_2_ emissions from aviation could increase by 300% by 2050. It is therefore very timely to address the problems of the synthesis of new navigation systems, which can contribute to more efficient use of airspace and a reduction in CO_2_ emissions.

The DME system has some drawbacks—including low resistance to interference [4,5,6,7,8,9,10,11,12,13,14,15]. Therefore, it is of practical importance that the task of optimizing the receiver of a DME system that operates under wide-band fluctuation interference conditions be addressed. Increased requirements to top quality of information transmission in very unfavorable noise conditions with strong interference as, e.g., in space communications or special aviation applications necessitate to seek new tendencies to improve the wireless electronic systems. It is interesting for us if it is possible to increase the accuracy and resistance of DME, which operates in conditions of wide-band interference type white noise. We approximate the useful signal DME by the Gaussian double pulse.

Several analyses performed on radio navigation systems [4,5,6,7,8,9,10,11,12,13,14,15] conclude that they operate under conditions of interference. The most typical forms of interference affecting its operation are termed as the wide-band fluctuation interference, narrow-band interference, and chaotic pulse interference. It has been proven [4,5,6,7,10,14] that the mentioned types of interference are substantially affecting the precision of measuring navigation parameters.

Tasks of the synthesis and analysis are based on the application of mathematical models of dynamic systems (object of navigation), signals, interference, and information processes [4,16,17,18,19,20,21,22,23,24,25]. The higher the precision of these a priori data, the higher the credibility of the results of the synthesis and analysis. Therefore, when developing mathematical models, it is appropriate to perform checks applying statistical tests [4,8], to determine the probability characteristics and based on them to decide whether the model is sufficiently precisely describing the essence of the process investigated.

In our research, we used the knowledge we gained when studying the possibility of using the telemetry method to navigate flying objects in the event of failure of satellite navigation systems signals. Substantial results of this research are presented in [5,24]. These works confirm that poor quality satellite systems signals have a significant impact on aviation safety. The results of the Telemetry Accuracy Assessment presented in [24] show that the determination of the position of a flying object operating in an aeronautical communication network is significantly affected by how accurately the distance is measured between that object and other network users with a known position. Therefore, we investigated how to synthesize accurate DMEs that would allow distance measurement with maximum accuracy. Issues of DME synthesis were addressed in [4]. In this study, algorithms for processing measurement signals when using binary carrier signals were derived. Algorithms presented in this work allowed estimating the potential accuracy of DME, but did not allow simulating the real accuracy of distance measurement by the DME system. 

In the studies of [9,15], the problems of improving the accuracy of aircraft positioning by navigational AIDS using Kalman filter are solved. The paper analyzes the implementation of alpha-beta-gamma filter in Kalman model representation to reduce noise in trajectory data of aircraft. It is stated that the sensor noise filtration of DME increases the accuracy of the position determination of the flying object.

The work [10] solves the DME performance problems. This article states that the resolution and fidelity of the base database and the accuracy of the DME ground system location surveys are a function of today’s 0.2 nm system error limit. Such a system error appears to be unacceptable for accurate positioning of the FO.

Possibilities of suppression of interference of the DME system based on the use of measurement signal estimation theory are given in [12]. The DME signal has a relatively high pulse power and can therefore cause GPS disturbance. This creates errors in GPS positioning of flying objects. In this paper, algorithms for suppressing DME interference based on a priori data about measurement signal are derived. Obviously, the use of DME in air traffic may cause interference of GPS signals. 

The paper [13] deals with a high accuracy DME pulse for alternative aircraft position and navigation. In the Federal Aviation Administration’s (FAA), the FAA stated that it would retain and expand the DME infrastructure to ensure resilient aircraft navigation capability during the event of a global navigation satellite system (GNSS) outage. However, the main drawback of the DME as a GNSS back up system is that it requires a significant expansion of the current DME ground infrastructure due to its poor distance measuring accuracy over 100 m. The paper presents a method for improving the accuracy of DME distance measurement using a new DME pulse shape. Thereby, so that the ranging error reduces by 36.0–77.3% when compared to the Gaussian and Smoothed Concave Polygon DME pulses, depending on the noise environment. 

The study [22] describes the Mosaic/DME navigation system. This proposal represents a new alternative for positioning, navigation, and timing in the event of a GNSS outage. In this paper, the proposed system is analyzed in terms of positioning accuracy using the Monte Carlo method. Based on the simulation, the authors conclude that the proposed system is suitable for determining the position of flying objects.

## 2. Model of DME Signal and Its Parameters

The DME input signal μ(t) is expressed as:

(1) μ(t) = S(X, t) + n(t)

where *S*(*X*, *t*)—effective signal; *n*(*t*)—wide band interference.

Wideband fluctuation interference is caused by the noise of the antenna, input circuits of the receiver, thermal radiation of the ground, cosmic noise, fluctuating character of the radio waves attenuation in the atmosphere, etc. The noises mentioned feature wide-band spectra. The spectral intensity of the process *n*(*t*) can be regarded as constant along the entire width of the receiver band. Consequently, interference *n*(*t*) is approximated by white, Gaussian noise with known characteristics [4,7,8]:

(2) $$E\left(n\left(t\right)\right)\;=\;0;\;E\left(n\left(t_{1}\right)\;\times \;n\left(t_{2}\right)\right)\;=\;N_{0}/2\;\times \;\delta \left(t_{2}\;-\;t_{1}\right)$$

where *N*_0_ is an intensity of the *n*(*t*) process. Relation (2) yields the white noise physically improbable results as its dispersion *D_n_* = ∞. In reality, it is impossible, as the width of the receiver band is final. 

The useful signal *S*(*X*, *t*) for radio navigation distance meter DME approximates Gaussian double pulse with a code interval of *τ_k_*. Individual pulses will be considered to be pulses of the quasi rectangular form [7]. In accordance with [4,18], the useful signal *S*(*X*, *t*) to be expressed as a sequence of pulses in shape:

(3) $$\begin{array}{cc} s\left(t,\;X\right)=A\left(t\right)\times & \{EXP[-\frac{\pi }{2\tau_{i}{}^{2}}\;{\left(t-t_{k}-t_{z}-\frac{2D\left(t\right)}{c}\right)}^{2}\\ & +EXP\left[-\frac{\pi }{2\tau_{i}{}^{2}}\right]{\left(t-t_{k}-t_{z}-\tau_{k}-\frac{2D\left(t\right)}{c}\right)}^{2}]\}\times \cos\left[\Omega_{0}t+\varphi \left(t\right)\right], \end{array}$$

where *A*(*t*)—pulse amplitude. Further, we assume that *A*(*t*) = *A*_0_ = const.; *D*(*t*)—inclined distance of flying object (FO) from overground responder; Ω_0_—useful signal frequency; *φ*(*t*)—random phase of useful signal; *τ_i_*—pulse length; *t_k_* = *t*_0_ + *k* × *T_i_*, *k* = 0, 1, 2, 3,....*n*—moment of transmitting the *k*th—pulse; *t*_0_—start of measurement; *t_z_*—time delay of signal in transmitter circuitry; *τ_k_*—code interval; *T_i_*—interrogation pulses period; *t_k_*—moment of first pulse transmission.

Signal parameters (3) will be equal: $\Omega_{0}=\;2\pi \times 1.10^{9}\;s^{-1},\;\tau_{k}\;=\;12\;\mu s.$

The model of the movement of a flying object is expressed by [4,7]:

(4) $$\frac{dD\left(t\right)}{dt}=\;V\left(t\right)=\;Vf\left(t\right)+\;V_{0},\;D\left(t_{0}\right)=\;D_{0}\;\frac{dVf\left(t\right)}{dt}=\;a\left(t\right),\;V_{f}\left(t_{0}\right)=\;V_{f0}\;\frac{dVf\left(t\right)}{dt}=\;a\left(t\right),\;V_{f}\left(t_{0}\right)\;=\;V_{f0}\;\frac{da}{dt}=\;-\alpha \times a\left(t\right)-ß.V_{f}\left(t\right)+\;{\left(2\times \alpha \times \sigma a^{2}\right)}^{0.5}\times n_{a}\left(t\right);\;a\left(t_{0}\right)\;=\;a_{0};\;\frac{dV_{0}}{dt}=0;\;V_{0}\left(t_{0}\right)\;=\;V_{00}$$

where *V_f_*(*t*)—fluctuation part of the radial component of track velocity; *V*_0_ = *E*(*V*(*t*))—mean of velocity *V*(*t*); α, β—are variable coefficients characterized spectral density of acceleration random changes which are determined by fluctuation component of wind velocity, object type, and conditions of its movement; *σ_a_*^2^ = *E* (*a*^2^(*t*))—variance of acceleration fluctuation which is depended upon atmosphere turbulence ability, motor thrust fluctuation, etc.; *n_a_*(*t*)—is a white Gaussian noise with zeroing mean and intensity equal to 1. Calculation of model coefficients of flying object movement (4) is stated in the literature [7].

The random phase of the measurement signal is expressed by [7]:

(5) $$\frac{d\varphi }{dt}=-\omega_{n}-\;\left(\frac{\Omega_{0}}{c}\right)\times \frac{dD\left(t\right)}{dt}+{\left(\frac{N\varphi }{2}\right)}^{0.5}$$

(6) $$\frac{d\omega_{n}}{dt}=-\gamma_{\omega }\times \omega_{n}+{\left(2\times \gamma_{\omega }\times \sigma_{\omega n}{}^{2}\right)}^{0.5}\times n_{\omega }\left(t\right)$$

where *ω_n_*—is the frequency deflection of supporting generator DME; *n_φ_*(*t*), *n_ω_*(*t*)—white Gauss noise with zero mean value and an intensity equaling to one; *γ_ω_*^−1^—is the time of correlation of processes *ω_n_*; *σ_ωn_*^2^—dispersion of the frequency fluctuation of supporting generator.

The calculation of model coefficients of the random phase is stated in the literature [7].

From Equations (1)–(6) it can be seen that status vector is comprised of five components:

(7) $$X^{T}\;=\;\left[D,\;V_{f},\;a,\;\varphi ,\;\omega_{n}\;\right]$$

and satisfies the systems of differential equations:

(8) $$\frac{dX}{dt}=F\times X\;+\;G\times N_{x}\left(t\right),\;X\left(t_{o}\right)\;=\;X_{0}$$

Matrix *F* and *G* of (5 × 5) dimensions consist of nonzero elements:

f_12_ = f_23_ = f_45_ = 1; f_32_ = −ß; f_33_ = −α; f_42_ = −Ω_0_/c; f_55_ = −*γ_ω_*; g_33_ = (2. α.*σ_a_*^2^)^0,5^; g_44_ = (*γ_ω_*. DF)^0,5^;

g_55_ = (2. *γ_ω_*.*σ_ωn_*^2^)^0,5^;

When calculating the matrix elements F and G it is assumed that the *S*(.) signal period is known. *Nx* = [0, 0, *n_a_*, *n_φ_*, *n_ω_*]^*T*^—a white Gaussian noise vector of zero mean value and of an intensity equal to one.

## 3. Algorithms of Optimum Filtering

If the realization of the process *μ* (*t*) at the time interval <0, *T*> is denoted as $\mu_{O}^{t}$, then all information on the implementation of the state vector *X*(*t*) contains a conditional (a posterior) probability distribution *p*(*X*, *t*|$\mu_{O}^{t}$) for the value of *X* under the condition that at the input of the optimum receiver there is a signal $\mu_{O}^{t}$ [8]. If we determine the quadratic loss function as the criterion of accuracy, which is expressed as:

*c*[*E*(*t*)] = [*X*(*t*) − X*(*t*)]. [*X*(*t*) − X*(*t*)]^*T*^, (9)

where *E*(*t*) = [*X*(*t*) − X*(*t*)], then the result of the optimal X*(*t*) measurement of the state vector *X*(*t*) is determined as the minimum of the a posterior mean error:

(10) $$\int c\left[X\left(t\right)-X^{*}\left(t\right)\right]\;p\;(X,\;t\;|\mu_{O}^{t})\;.\;\mathrm{dX}\;=\;\min$$

For the quadratic loss function that corresponds to the minimum mean squared error criterion, the quality of the measurement is characterized by a [7,8] matrix of second-order a posteriori central moments of filtration errors K(t):

(11) $$K(t)\;=\;\int [X\left(t\right)-X^{*}\left(t\right)].\;[X(t)-X^{*}(t){]}^{T}.\;p\;(X,t\;|\mu_{O}^{t})\;.\;\mathrm{dX}\;=\;\min$$

In accordance with relation 9, the following applies:

(12) $$X^{*}(t)\;=\;\int_{-\infty }^{+\infty }X.\;p\;(X,t\;|\mu_{O}^{t})\;.\;\prod_{i=1}^{m}dX_{i}$$

The criterion of optimality will be the minimum of the mean square error. Then, the measurement results given by Equation (12) represent the a posteriori mean value of the vector *X*(*t*). To express *p* (*X*,*t* |$\mu_{O}^{t})$ we need a relation for a priori probability density *p_a_* (*X* (*t*)). The a priori probability density *p_a_* (*X*(*t*)) of the *X*(*t*) process complies with the Fokker–Plank–Kolmogorov Equation [5]:

(13) $$\frac{dp_{a}\;\left(X,t\right)}{dt}=L(p_{a}(X,\;t))$$

where L(*p_a_*(*X*, *t*)) is the Fokker–Plank–Kolmogorov operator.

Derivation of general equations for the a posteriori probability density *p* (*X*, *t* |$\mu_{O}^{t}$) is given, for example, in [7,8] based on the use of the Bayes formula. Furthermore, it is shown that if the measurement signal *S* (*X*, *t*) is received in the background of normal noise *n*(*t*), then the a posterior probability density *p* (*X*, *t* |$\mu_{O}^{t}$) satisfies the differential equation of Stratonovič [8].

To obtain X*(*t*) it is necessary to find a solution for the a posteriori probability density *p* (*X*,*t* |$\mu_{O}^{t}$). The exact solution of this equation is very complicated and therefore, different methods based on approximations of *p* (*X*,*t* |$\mu_{O}^{t}$) are used in practice. In [7,8], solutions are given for *p* (*X*,*t* |$\mu_{O}^{t}$) in case the signal *S* (*X*, *t*) depends on the multivariate Markov process *X*(*t*). The abovementioned solutions are general for processing the signal *S* (*X*, *t*) received in the background of white noise *n*(*t*). In our research, we were looking for a solution for processing DME signals received in the background of white noise. We used the Gaussian approximation methods [8], the continuous linearization method [5], and the method of large and small parameter [7]. Based on this, we derived algorithms for processing DME signals under noise interference conditions *n*(*t*).

In accordance with Equations (1) to (13), the algorithms of optimum nonlinear DME signals filtration have the forms [4,7,8,18]:

(14) $$\frac{dX^{*}}{dt}=F\times X^{*}\left(t\right)\;+\;K\left(t\right)\times F_{1}\left(X^{*}\left(t\right)\right)\times \left[\mu \left(t\right)\;-\;S\left(X^{*}\left(t\right)\right)\right],\;X\left(t_{0}\right)=X_{0}$$

(15) $$\frac{dK}{dt}=\;F\times K\left(t\right)+K\left(t\right)\times F^{T}+Q-K\left(t\right)\times F_{k}\left(X^{*}\left(t\right)\right)\times K\left(t\right);\;K\left(t_{0}\right)\;=\;K_{0}$$

where matrix:

(16) $$F_{1}\left(X^{*},t\right)=\;\left[\frac{dS\left(X^{*},t\right)}{dX_{1}^{*}},\;0,0,\;\frac{dS\left(X^{*},t\right)}{dX_{4}^{*}},0\right]$$

matrix:

(17) $$F_{k}\left(X^{*},t\right)=\left|\begin{array}{ccc} {\left(\frac{dS\left(X^{*},t\right)}{dX^{*}{}_{1}}\right)}^{2} & \ldots & \frac{d^{2}S\left(X^{*},t\right)}{dX^{*}{}_{1}dX^{*}{}_{5}}\\ \vdots & \vdots & \vdots \\ \frac{d^{2}S\left(X^{*},t\right)}{dX^{*}{}_{1}dX^{*}{}_{5}} & \ldots & {\left(\frac{d^{2}S\left(X^{*},t\right)}{d^{2}X^{*}{}_{5}}\right)}^{2} \end{array}\right|$$

**Figure 1:**
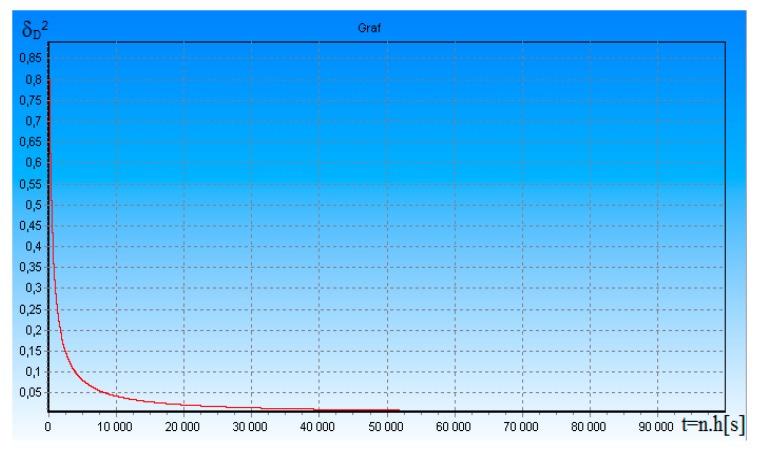
Dependence of the δ_D_^2^ of standardized measurement error of the slant range D(t) between FO (Flying Object) and distance-measuring equipment (DME), on time t.

**Figure 2:**
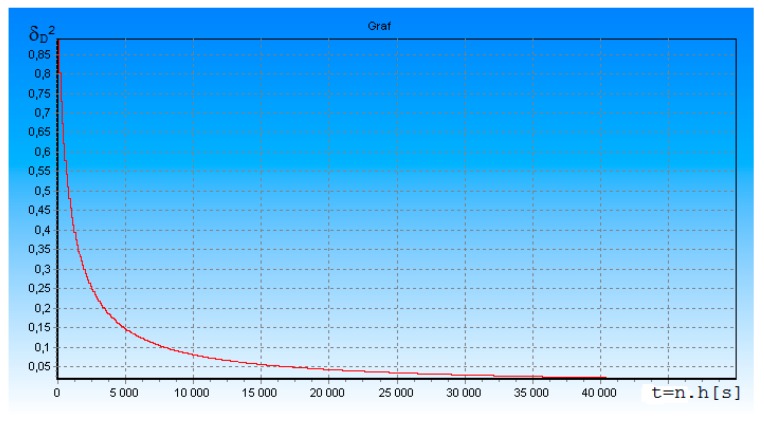
Dependence of the δ_D_^2^ of standardized measurement error of the slant range D(t) between FO (Flying Object) and distance-measuring equipment (DME), on time t = 0 to 0.05 s.

**Figure 3:**
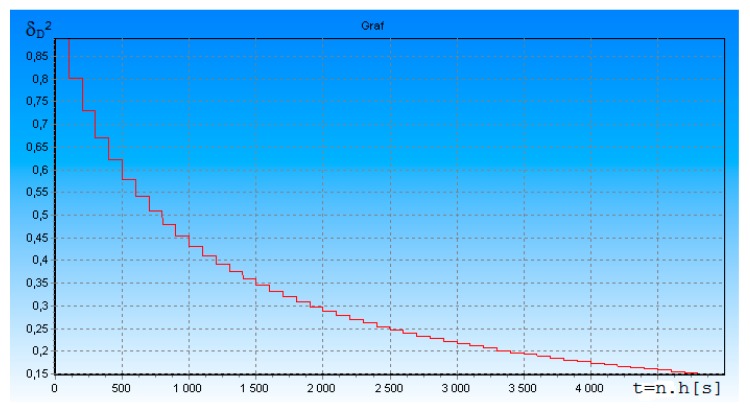
Dependence of the δ_D_^2^ of standardized measurement error of the slant range D(t) between FO (Flying Object) and distance-measuring equipment (DME) in the transition process. Time t = 0 to 0.05 s.

**Figure 4:**
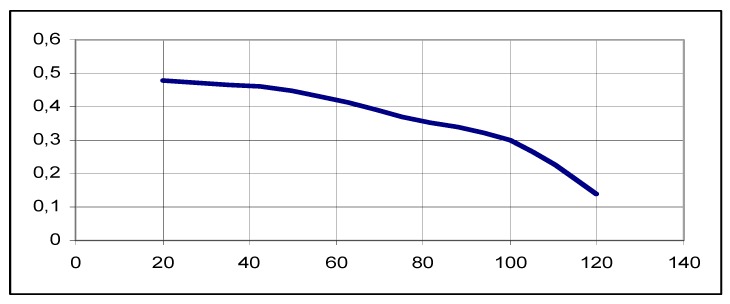
Dependence of the standardized a posteriori dispersion δ_D_^2^ on the number of measured pulses received in the transition process.

**Figure 5:**
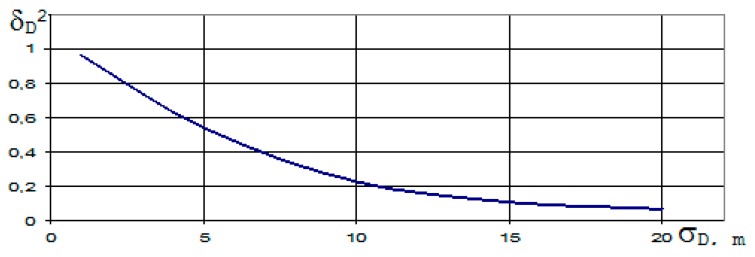
Dependence of the a posteriori standardized dispersion δ_D_^2^ (t) on a priori dispersion σ_D_ in the transition process for t = 0.06 s [DPBA].

**Figure 6:**
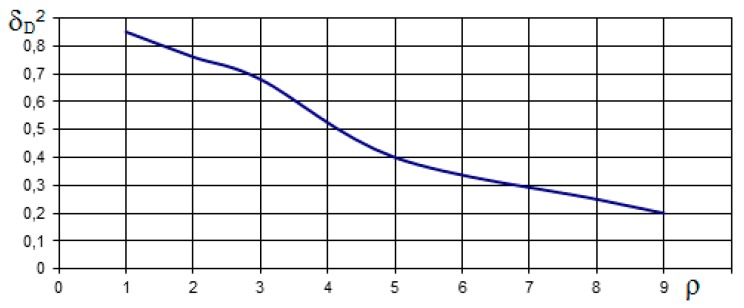
Dependence of the a posteriori standardized dispersion δ_D_^2^(t) on the useful signal-to-noise ratio ρ in the transition process [DPBA].

**Figure 7:**
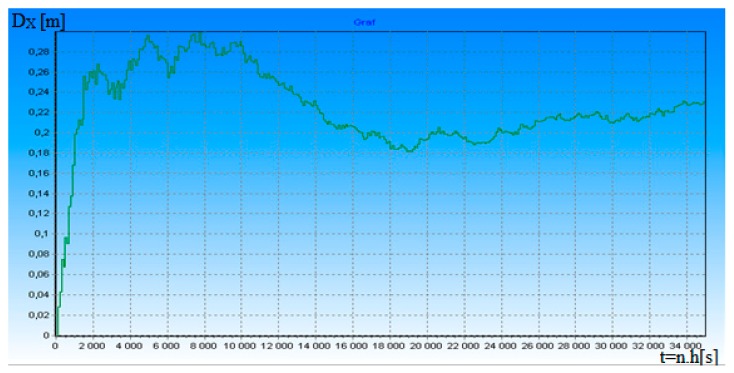
Measurement distance dependence error D_X_ on time t for ρ = 2.

**Figure 8:**
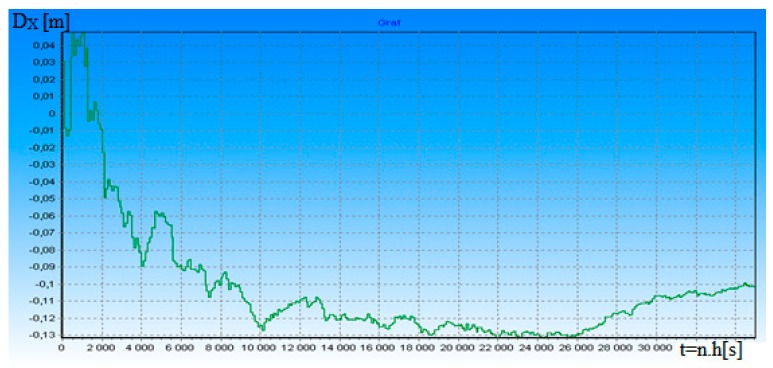
Measurement distance dependence error D_X_ on time t for ρ = 12.

The * symbol indicates the measured signal parameter. *K*—a posteriori dispersion of the filtration errors of the status vector. Equations (14) and (15) determine the structure, accuracy, and resistance to wide-band band interference of DME, which has the best (depending on the simplifications adopted in deriving relations (14) and (15) the accuracy characteristics. Based on the derived algorithms (14) and (15), we simulated the measurement of navigation parameters by the DME system. The ability of the DME receiver to operate under wide-band fluctuation conditions was verified through modeling. By calculating the relation (15) and substituting the covariance matrix of the a posteriori error filtering of the signal parameters (3) K(t) into Equation (14), it is possible to model the process of measuring the distance of the flying object from DME using a PC modeling, thus allowing the dependence of DME characteristics on the change of input signal parameters, wide-band fluctuation failure, and flight dynamics of the flying object to be determined. Modeling results can be used in the practical construction of the DME receiver [4,21,22]. The created algorithms allow us to determine the sensitivity of DME to the intensity of the interference signal. The simulation results are shown in Figure 1, Figure 2, Figure 3, Figure 4, Figure 5, Figure 6, Figure 7 and Figure 8. The matrix K(t) is expressed in a standard form:

(18) $$\delta_{ij}\;=\;K_{ij}{\sigma_{i}}^{-1}\times \sigma_{j}{}^{-1}$$

where *σ_i_*^−1^.*σ_j_*^−1^—a priori variance of signal parameters (3); *i* = {1,2,.......5}; *j* = {1,2,......5}.

The initial conditions for matrices K(t) have the form: *δ_ii_* = 1; *δ_ij_* = 0.

In simulations, the initial conditions were determined to correspond to the physical sense of the task as follows [1,4,15]:

α = 0.5 s^−1^; β = 3.5 × 10^−2^ s^−2^; σ_F_ = 0.3 rad; D_F_ = 10^−3^; σ_D_ = 15.0 m; σ_V_ = 0.1 ms^−1^; σ_a_ = 1.0 m s^−2^;

γ_ω_ = 300.0 s^−1^; σ_ωn_ = 1 − 10^3^ s^−1^; f_0_ = 1.0 − 10^9^ s^−1^;

The useful signal-to-noise ratio was of the range 1–12. The initial conditions for the state vector: X^T^ = [1000; 10; 5; 0; 0]; V_00_ = 100 ms^−1^. 

## 4. Discussion

After deriving the optimal DME algorithms, we performed a simulation of the potential (maximum) accuracy of DME according to Equation (15). The simulation results are shown in Figure 1. Figure 1 shows the dependence of standardized a posteriori dispersion δ_D_^2^ of error measurement of the slant range D(t) between FO and DME, on time t = n × h [s], where h = 1 × 10^−5^ is integration step; n is the number of integration steps. The useful signal-to-noise ratio ρ was equal to 10. T_i_ = 1.66 × 10^−3^—interrogation pulses period.

It is clear from Figure 1 that the measurement errors are relatively large at the beginning of the measurement. After several measurements, the errors have stabilized to a minimum value. The time that elapses until the beginning of the measurement to steady state is referred to as the transition process. In this case, the length of the transition process is approximately 0.03 s. Approximately within 0.3 s the transition process is practically terminated and potential accuracy of DME for ρ = 10 and T_i_ = 166 × 10^−3^ is equal to: (K_DD_)0.5 = (δ_D_^2^ (t) × σ_D_^2^)0.5 = 2.25 m. The length of the transition process will depend on the conditions in which the DME operates. It is especially true from the useful signal-to-noise ratio ρ and the interrogation pulses of T_i_ period. Therefore, we have checked the sensitivity of the algorithm (15) to change these parameters.

Figure 2 shows the dependence of the standardized posterior dispersion δ_D_^2^ of measurement error of the slant range D(t) between FO and DME, on time t = n × h [s], where h = 1 × 10^−5^ is integration step; n is the number of integration steps. The useful signal-to-noise ratio ρ was equal to 5. T_i_ = 1.0 × 10^−3^—interrogation pulses period. From the simulation results, we can see that the change of simulation parameters also changes the character of the transition process of DME measurement. The length of the transient process is practically unchanged, but the measurement errors are more significant at the beginning of the measurement, although we have reduced the measurement pulse period. This is due to a reduction in the useful signal-to-noise ratio ρ. Next, we have checked how the receiver works in time intervals when there is no measuring signal at its input. The simulation results are shown in Figure 3. 

Figure 3 shows the dependence of the standardized posterior dispersion δ_D_^2^ of measurement error of the slant range D(t) between FO and DME, on time t = n × h [s] = 0.05 s., where h = 1 × 10^−5^ is an integration step; n is the number of integration steps. The useful signal-to-noise ratio ρ was equal to 5. T_i_ = 1.0 × 10^−3^—interrogation pulses period.

The simulation results in Figure 3 shows that in the transient process, the measurement error (decrease δ_D_^2^) is significantly reduced after receiving the measurement signal. The measurement error does not change substantially in the spacing between the measurement signals. This is because the receiver extrapolates the measured navigation parameter value, and therefore the drift error increases only slightly.

Figure 4 shows the dependence of the standardized a posteriori dispersion δ_D_^2^(t) on the number of measured pulses received. The graph on Figure 4 shows that δ_D_^2^(t), in transition mode, depends on the number of received DME on-board transponder pulses.

The more measurement pulses the DME receiver receives, the higher the accuracy of radial distance measurement D(t)—i.e., the smaller the interrogation pulses period, the higher the measurement accuracy. If there is no useful signal at the receiver input, the receiver performs extrapolation of distances D(t) according to algorithms (14) and (15).

Figure 5 shows the dependents of δ_D_^2^ from σ_D_. It is evident from the figures that the transition process is shortened as σ_D_ increases and the potential accuracy of distance measurement deteriorates. The mean square deviation (K_DD_)^0.5^ = (δ_D_^2^(t) × σ^2^_D_)^0.5^ at σ_D_ = 5 m equals 2.7 m and at σ_D_ = 10 m equals 3.3 m.

Figure 6 shows the dependence of the a posteriori standardized dispersion δ_D_^2^(t) on the useful signal-to-noise ratio ρ in the transition process for t = 0.012 s. When increasing the ratio ρ by nine times, δ_D_^2^(t) filtration accuracy will be increased by 4.5 times and it explains the fact that DME is, to a certain degree, resistant to the fluctuation of the useful signal-to-noise ratio ρ. Figure 7 and Figure 8 depict the dependence of the difference in D_X_ distance from the ρ ratio in transition mode. D_X_ = D(t) − D*(t), where D(t) is the modeled distance and D*(t) is the measured distance in accordance with algorithms 14 and 15.

Modeling confirmed that changing the ρ in intervals of 2 to 12 changes the potential accuracy of DME. The results of calculating the potential accuracy and modeling the absolute error of D_X_ distance measurement by suboptimal DME confirm that the particular method of assessing the potential and real accuracy of radio navigation systems operating under wide-band fluctuation interference conditions is applicable in practice.

## 5. Conclusions

The aim of our research was to point out that without new technologies, environmental problems in air transport are unsolvable. One of the negative impacts of air transport on the environment is the emission of harmful substances into the air [25]. The European Environmental Report on Air Transport 2019 states that the impact of aviation on climate change, noise, and air quality is increasing and thus affects the health and quality of life of European citizens. Therefore, it is current to design precision navigation systems that allow optimizing flight trajectories and thereby reducing harmful emissions to the environment [23]. With the increasing use of aviation, the demands on the accuracy and reliability of navigational tasks, radio navigation equipment, and systems are also increasing [19].

As mentioned in the introduction, in our research we used the knowledge we gained when studying the possibility of using the telemetry method to navigate flying objects in the event of failure of satellite navigation systems signals. Substantial results of this research are presented in [5,24]. These works confirm that poor quality satellite systems signals have a significant impact on aviation safety. The results of the Telemetry Accuracy Assessment presented in [24] show that the accuracy of the positioning of a flying object operating in an aviation communication network is significantly affected by the accuracy of the distance measurement between that object and other network users of known position.

In this work, the primary attention is paid to methods of evaluation of accuracy and resistance of the DME system against the interference, using the theory of optimal nonlinear filtering. Findings from the operation of navigation systems confirm that their input signals are degraded by interference of various kinds, which causes deterioration in the accuracy of the systems themselves. Therefore, when optimizing and designing new navigation systems, it is necessary to use statistical methods of analysis and synthesis that are based on a statistical understanding of the processes taking place in these systems. This approach makes it possible to optimize the systems and significantly increase their accuracy and interference resistance.

The created algorithms were simulated using the Delphi software. This made it possible to speed up the execution of the calculation, to increase the accuracy of the results, but in particular to significantly increase the possibilities of simulations. The results of the work extend the possibilities of using navigation measurement parameter modeling processes that allow us to a certain extent to predict the characteristics of the proposed RNS before its immediate construction. These results can be used in the design of precision navigation systems that are highly resistant to interference. Accurate navigation systems allow us to optimize the trajectory of flying objects and thus solve environmental problems in aviation and their impact on public health.

The results of the calculations of potential accuracy and simulation of distance D(t) measurement between FO and DME by means of an optimum receiver of DME according to algorithms (14) and (15), confirm that the presented method of assessment of the potential and real accuracy of DME is applicable in practice when performing synthesis of new DME. Assuming the availability of a priori data on existing radio navigation systems and the signals they use for navigation measurements, the present method can be used to analyze these systems for their accuracy and interference immunity. The modeling results show that the interference [24] has a significant impact on the accuracy of the navigation parameter measurement.

The main benefits of our research are the creation of algorithms for processing measured signals by the DME system and their verification by simulation. The created algorithms determine the architecture of the DME receiver. On the basis of these algorithms, it is possible to create a block and, consequently, also a basic connection of the receiver. Using modeling, we verified the potential accuracy of the proposed DME. We also examined the sensitivity of algorithms to change DME operating parameters. We compared our results with the results of other published works in this field. It should be noted here that the algorithms for processing the signal measurements of navigation systems are mostly not published. They are strictly guarded by companies that manufacture navigation systems.

In accordance with algorithms (14) and (15), we investigated the accuracy of our proposed DME. From the results shown in Figure 1, Figure 2, Figure 3 and Figure 4, it can be seen that approximately within 0.3 s, the transition process is practically terminated and the potential accuracy of DME for ρ = 5 is equal to: (K_DD_)^0.5^ = (δ_D_^2^ (t) × σ_D_^2^)^0.5^ = 2.25 m. The graph on Figure 3 and Figure 4 shows that δ_D_^2^(t) in transition mode depends on the number of received DME on-board transponder pulses. The more response pulses received over a given period of time, the higher the accuracy of radial distance measurement D(t). If these results are compared to the DME accuracy requirements given in [10], the accuracy of the rangefinder proposed by us is much better. 

From the DME system navigation parameter measurement modeling, it is obvious that the standardized covariance coefficients depend on the parameters of the model created and the signal-to-noise ratio at the receiver input. The calculations confirmed that after the transition process the DME receiver operates in quasi-stationary mode—thus making it possible to substantially simplify the structure of quasi-optimal DME, thereby shortening the time it takes to process measurement signals and evaluate measurement results.

We also examined the sensitivity of algorithms (14) and (15) to change a priori data. It is evident from Figure 5 that the transition process is shortened as σ_D_ increases and the potential accuracy of distance measurement deteriorates. The mean square deviation (K_DD_)^0.5^ = (δ_D_^2^(t) × σ^2^_D_)^0.5^ at σ_D_ = 5 m equals 2.7 m and at σ_D_ = 10 m equals 3.3 m. 

It is evident from Figure 6 that when increasing the ratio ρ by nine times, δ_D_^2^(t) filtration accuracy will be increased by 4.5 times and it explains the fact that DME is, to a certain degree, resistant to the fluctuation of the useful signal-to-noise ratio ρ. Modeling confirmed that the better the measurement signal we have (the higher the ρ value), the better the measurement accuracy.

According to algorithms (14) and (15), we modeled the real accuracy of DME distance measurement. Modeling confirmed that changing the ρ in intervals of 2 to 12 changes the potential accuracy of DME. Distance errors varied between 0.3 and −0.13 m. Work [13] deals with a high accuracy DME pulse for alternative aircraft position and navigation. The paper introduces a method to improve DME distance measuring accuracy by using a new DME pulse shape. Thereby, so that the ranging error reduces by 36.0–77.3% (64.0–22.7 m) when compared to the Gaussian and Smoothed Concave Polygon DME pulses, depending on the noise environment. Our results (measurement error 0.3 and −0.13 m) are much better. It is necessary to say here that the realization of DME hardware according to algorithms (14) and (15) can be a problem if we want to realize the real-time measurement of measurement signals. Due to the very high operating frequency of the DME system, processing of its specific signals according to algorithms (14) and (15) requires high-speed signal processors. To our knowledge, such processors are currently unavailable. Therefore, the practical implementation of the hardware algorithms are simplified. However, this will reduce the accuracy of the measurement of the navigation parameter.

The results of the investigation confirm that the application of statistical methods in the evaluation and design of radio navigation systems makes it possible to assess the impact of interference on their accuracy in determining the position of flying objects. New approaches to the design of navigation systems can contribute to maintaining the trend of strong air transport growth and reducing its environmental impact.
